# Mediterranean Diet Adherence, Physical Activity, and Advanced Glycation End Products in Complex PTSD: A Comprehensive Examination of Lifestyle and Cardiovascular Risk in War Veterans

**DOI:** 10.3390/nu16111791

**Published:** 2024-06-06

**Authors:** Zivana Puljiz, Marko Kumric, Tonka Borovina Marasovic, Tonci Mastelic, Mihaela Rakusic, Goran Pavela, Andelko Beg, Trpimir Glavina, Marin Mornar, Daniela Supe Domic, Marino Vilovic, Jurica Zucko, Tina Ticinovic Kurir, Josko Bozic

**Affiliations:** 1 Laboratory for Bioinformatics, Faculty of Food Technology and Biotechnology, University of Zagreb, 10000 Zagreb, Croatia; zivana.puljiz@servier.com (Z.P.); jzucko@pbf.hr (J.Z.); 2 Department of Pathophysiology, University of Split School of Medicine, 21000 Split, Croatia; marko.kumric@mefst.hr (M.K.); marino.vilovic@mefst.hr (M.V.); tticinov@mefst.hr (T.T.K.); 3 Laboratory for Cardiometabolic Research, University of Split School of Medicine, 21000 Split, Croatia; 4 Department of Psychiatry, University Hospital of Split, 21000 Split, Croatia; tborovina@kbsplit.hr (T.B.M.); toncimastelic@hotmail.com (T.M.); mrakusic@kbsplit.hr (M.R.); gpavela@kbsplit.hr (G.P.); abeg@kbsplit.hr (A.B.); tglavina@kbsplit.hr (T.G.); 5 Department of Psychiatry, University of Split School of Medicine, 21000 Split, Croatia; 6 Department of Pharmacology, University of Split School of Medicine, 21000 Split, Croatia; marin.mornar@mefst.hr; 7 Department of Health Studies, University of Split, 21000 Split, Croatia; daniela.supe.domic@ozs.unist.hr; 8 Department of Medical Laboratory Diagnostics, University Hospital of Split, 21000 Split, Croatia; 9 Department of Endocrinology, Diabetes and Metabolic Disorders, University Hospital of Split, 21000 Split, Croatia

**Keywords:** Mediterranean diet, post-traumatic stress disorder, lifestyle, advanced glycation end products, cardiovascular risk

## Abstract

As accumulated evidence suggests that individuals with post-traumatic stress disorder (PTSD) encounter earlier and more frequent occurrences of cardiovascular diseases, the aim of this study was to ascertain the differences in lifestyle and cardiovascular risk between PTSD and complex PTSD patients. We enrolled 137 male war veterans with PTSD (89 had complex PTSD). The diagnosis was established based on 11th revision of International Classification of Diseases (ICD-11), and cardiovascular risk was estimated by the measurement of advanced glycation end products. Adherence to Mediterranean diet (MD) was lower in the complex PTSD group (2.2% vs. 12.5%, *p* = 0.015). Accordingly, patients with complex PTSD had lower healthy lifestyle scores in comparison to PTSD counterparts (50.6 ± 9.7 vs. 59.6 ± 10.1, *p* < 0.001), and a positive association was noted between MD adherence and a healthy lifestyle (r = 0.183, *p* = 0.022). On the other hand, differences were not noted in terms of physical activity (*p* = 0.424), fat % (*p* = 0.571) or cardiovascular risk (*p* = 0.573). Although complex PTSD patients exhibit worse adherence to MD and lower healthy lifestyle scores, these differences do not seem to impact physical activity, body composition, or estimated cardiovascular risk. More research is needed to clarify if this lack of association accurately reflects the state of the PTSD population or results from insufficient statistical power.

## 1. Introduction

Due to the outbreak of the civil war in the early 1990s, the incidence of post-traumatic stress disorder (PTSD) has significantly increased in Croatia [1]. Beyond its psychiatric implications, accumulated evidence suggests that individuals with PTSD face earlier and more frequent occurrences of cardiovascular diseases (CVD), identifying PTSD as an independent risk factor for CVD [2].

This association is complex, involving mediation by factors such as inflammation, oxidative stress, obesity, and lifestyle elements like poor diet and reduced physical activity [3]. Studies consistently demonstrate that individuals with PTSD are at higher risk of obesity, engage in less physical activity, and are avid smokers [4,5]. Additionally, recent research has identified a correlation between PTSD symptoms and adherence to healthy dietary patterns, notably the Mediterranean diet (MD) [6,7]. This link is significant considering the strong advocacy by the American Diabetes Association (ADA) for following the MD due to its positive impact on glycemic control and cardiovascular risk factors, potentially elucidating adverse cardiovascular outcomes in individuals with PTSD [8].

Moreover, existing data underscore oxidative stress and inflammation as mediators of CVD, with extensively discussed interactions between inflammation, oxidative stress, and psychological stress [9]. Heightened oxidative stress leads to the formation of advanced glycation end products (AGEs), predominantly accumulating in low-metabolism tissues [10]. Besides exacerbating oxidative stress, the accumulation of AGEs in the skin serves as a valuable diagnostic biomarker for CVDs [11,12].

Given the interplay between lifestyle aspects and cardiovascular risk in PTSD, the primary objective was to ascertain the differences with respect to adherence to the MD and cardiovascular risk evaluated through the tissue levels of AGEs between male war veterans diagnosed with PTSD and complex PTSD. Additionally, a secondary aim was to explore the association between MD adherence, body composition, and a healthy lifestyle in these patients. 

## 2. Materials and Methods

### 2.1. Study Design and Participants

This research employed a cross-sectional design conducted at the Psychotrauma Center of the Department of Psychiatry, University Hospital of Split, from February 2023 to January 2024. Participation in this study was entirely voluntary, and each participant signed the informed consent form after a comprehensive explanation of this study’s procedures. Ethical approval was obtained from the Ethical Committee of the University Hospital of Split (Class: 003-08/23-03/0015; No: 2181-198-03-04-23-0005; Date: 31 January 2023), ensuring compliance with the ethical principles outlined in the Helsinki Declaration.

The primary inclusion criteria encompassed male individuals aged between 45 and 65 years old, actively involved in the Croatian War of Independence (1991–1995) and diagnosed with PTSD according to the 11th revision of the International Classification of Diseases (ICD-11) [13]. In line with the ICD-11 criteria, patients were categorized into PTSD or complex PTSD groups based on the presence of disturbances of self-organization (DSO) symptoms [13]. Specifically, the confirmation of a complex PTSD diagnosis required the endorsement of at least one symptom within each of the three DSO symptom clusters: emotional dysregulation, interpersonal difficulties, and negative self-concept, in addition to meeting the diagnostic criteria for PTSD [13]. To endorse a DSO symptom or functional impairment item, a score of ≥2, indicating ‘Moderately’, was necessary for the respective question. The diagnosis of complex PTSD was established using the International Trauma Questionnaire (ITQ), a validated assessment tool for evaluating both ICD-11 PTSD and complex PTSD [14]. Exclusion criteria for the study encompassed female gender; BMI > 35 kg/m^2^; significant chronic heart, kidney, or gastrointestinal diseases; diabetes mellitus; acute infections; recent malignancies; subjects that recently underwent a weight loss regime; and subjects with psychological disorders unrelated to PTSD. The recruitment of all participants was conducted through the aforementioned Psychotrauma Center.

### 2.2. Procedures

Upon signing the written informed consent form, each participant underwent two clinical examinations conducted by subspecialists in endocrinology and psychiatry, respectively. These examinations recorded basic anthropometric data including age, sex, body weight, height, waist circumference, and hip circumference for each participant. Using standard formulas, the body mass index (BMI) and the waist-to-hip ratio (WHR) were calculated based on the collected measurements. Furthermore, participants underwent body composition analysis by utilizing the Tanita DC360-S bioimpedance scale (Tanita, Tokyo, Japan). Key parameters of interest included body fat percentage, visceral fat quantity, muscle mass, and phase angle. 

In addition to the ITQ, participants were provided with a survey encompassing inquiries about dietary attitudes and three validated questionnaires: the Mediterranean Diet Serving Score (MDSS), the International Physical Activity Questionnaire Short Form (IPAQ-SF), and the Fantastic Lifestyle Questionnaire (FLQ).

The MDSS measures adherence to the Mediterranean diet based on recommended intake of 14 food groups outlined in the Mediterranean dietary pyramid [15]. Participants’ responses were scored based on their frequency of consuming these food groups using the MDSS scale, generating individual total scores ranging from 0 to 24. A score ≥ 14 indicated appropriate adherence to the Mediterranean diet. Marendic et al. assessed the reliability and validity of the Croatian version of the MDSS questionnaire, consisting of 14 items evaluating adherence to this dietary pattern [16].

The IPAQ-SF is a questionnaire previously verified in the Croatian language that evaluates self-reported activity across various intensity levels, including vigorous-intensity activities, moderate-intensity activities, walking, and sitting [17]. To align with observational study recommendations from the IPAQ-SF authors, we utilized the “last 7 days recall” version [17]. MET (metabolic equivalent of task) minutes per week scores were computed from IPAQ-SF data using specific formulas [17]: walking MET-min/week = 3.3 × walking minutes × walking days; moderate MET-min/week = 4.0 × moderate activity minutes × moderate days; vigorous MET-min/week = 8.0 × vigorous activity minutes × vigorous days; and total MET-min/week = sum of walking, moderate, and vigorous MET-min/week scores.

The FLQ, a questionnaire recognized for its robust reliability in previous studies, was employed to evaluate dimensions of a healthy lifestyle encompassing physical, social, and psychological aspects [18]. Initially developed by Wilson and Ciliska, the FLQ was later enhanced by the Canadian Society for Exercise Physiology to offer a more comprehensive individual assessment [18]. Featuring 25 questions, this questionnaire explores an individual’s behavior over the past month across nine distinct domains (Family/Friends, Activity, Nutrition, Tobacco/Toxins, Alcohol, Sleep/Seatbelt/Stress/Safe Sex, Type of Behavior, Insight, Career). Most items utilize a Likert scale with five response options, while two are dichotomous. Upon computation, the final score ranges from 0 to 100 points, with higher scores indicating a more health-conscious lifestyle. Subsequently, based on the scores, participants were categorized into five groups: 0–34 points indicating “needs improvement”, 35–54 points representing “fair”, 55–69 points denoting “good”, 70–84 points reflecting “very good”, and 85–100 points signifying “excellent”. As the FLQ was not validated in the Croatian language, it was translated for this purpose using a back-translation technique by an English language expert. The internal consistency of the FLQ in our sample was rendered acceptable, with the Cronbach alpha coefficient being 0.74.

After completion of the questionnaires, a peripheral blood sample was drawn from the forearm vein of each participant, collecting a maximum of 10 mL of blood per individual. Routine patient testing parameters were assessed from peripheral blood, including lipid profile (total cholesterol, low-density lipoprotein (LDL) cholesterol, high-density lipoprotein (HDL) cholesterol, triglycerides) and HbA1c. Lipid profile was analyzed via an enzymatic, colorimetric method on Cobas 701/702 (Roche Diagnostics GmBH, Mannheim, Germany). HbA1c levels were analyzed using High-Performance Liquid Chromatography (HPLC) using the Tosoh G8 HPLC Analyzer (Tosoh Bioscience, San Francisco, CA, USA). All blood samples underwent analysis at the same certified institutional biochemical laboratory following standard operating procedures. The analysis was conducted by an experienced biochemist blinded to participant group allocation.

The estimation of cardiovascular risk relied on tissue levels of AGEs. The assessment of AGEs was carried out non-invasively using an autofluorescence skin method with the AGE-Reader SU device (DiagnOptics Technologies BV, Groningen, The Netherlands). This device utilizes the characteristic fluorescence of specific AGEs to determine their accumulation in the skin. This method strongly correlates with measuring AGEs in a skin biopsy sample from the same site [19]. Measurements were taken on the right forearm, a standard measuring point, and the mean value of three consecutive measurements was used for analysis. Participants were categorized into four groups based on cardiovascular risk, derived from the correlation of the obtained result with age (no risk, limited risk, increased risk, definite cardiovascular risk). Importantly, this method is observer-independent and demonstrates an intrapersonal coefficient of variation of less than 5% [20].

### 2.3. Statistical Analysis

MedCalc Statistical Software version 20.113 (MedCalc Software BV, Ostend, Belgium) and Prism 8 version 8.01 (GraphPad, La Jolla, CA, USA) were utilized for the statistical analysis and graphical representation of data. Quantitative data were expressed as either mean ± standard deviation (SD) or median and interquartile range (IQR), while qualitative data were presented as whole numbers and percentages. The Shapiro–Wilk test was employed to assess the normality of data distribution. The comparison of qualitative variables was conducted using the chi-squared (χ^2^) test. For comparing quantitative variables, Student’s *t*-test for independent samples and the Mann–Whitney U test were utilized. In cases with 3 or more subgroups, the Kruskal–Wallis with post hoc Conover test was employed. Spearman’s rank correlation analysis was utilized to explore correlations between variables of interest. Statistical significance was set at *p* < 0.05 for all comparisons.

## 3. Results

A total of 137 male patients were included in the present study. Except for the fact that PTSD patients were more likely to be retired (*p* = 0.007) and spent more time at war than those with complex PTSD (*p* = 0.034), no other differences in baseline characteristics were found between the groups of interest (Table 1).

### 3.1. Patients with Complex PTSD Are Less Adherent to the Mediterranean Diet

**Figure 1:**
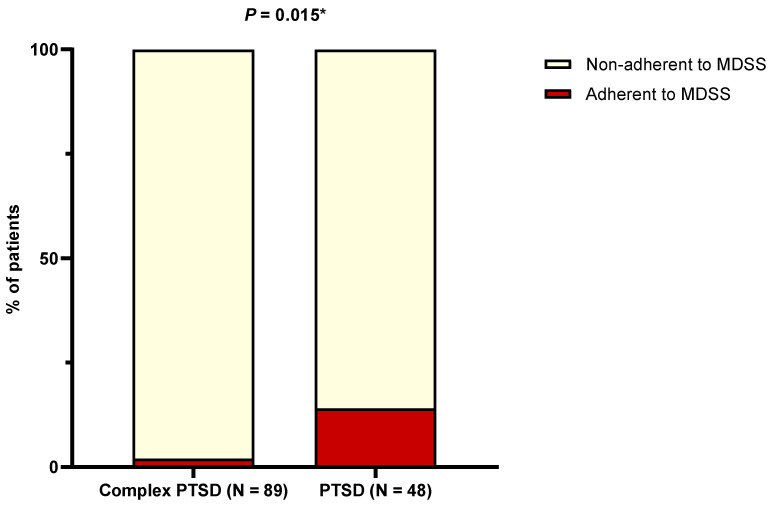
Difference in the proportion of patients who were adherent to the Mediterranean diet with respect to the presence of the complex form of PTSD. Abbreviations: MDSS: Mediterranean Diet Serving Score; PTSD: post-traumatic stress disorder. * Chi-squared test.

According to MDSS results, only 5.8% of patients were considered adherent to MD. Notably, adherence was significantly lower in complex PTSD patients in comparison to PTSD patients (2.2% vs. 12.5%, *p* = 0.015) (Figure 1). A comparison of adherence to each of the 14 individual food groups is presented in Table 2. Overall, patients with complex PTSD were less adherent to vegetables than patients with PTSD (*p* < 0.001), while the difference in adherence to white meat was marginally non-significant (*p* = 0.054).

### 3.2. AGE-Based CV Risk Does Not Vary between Patients with PTSD and Those with Complex PTSD

**Figure 2:**
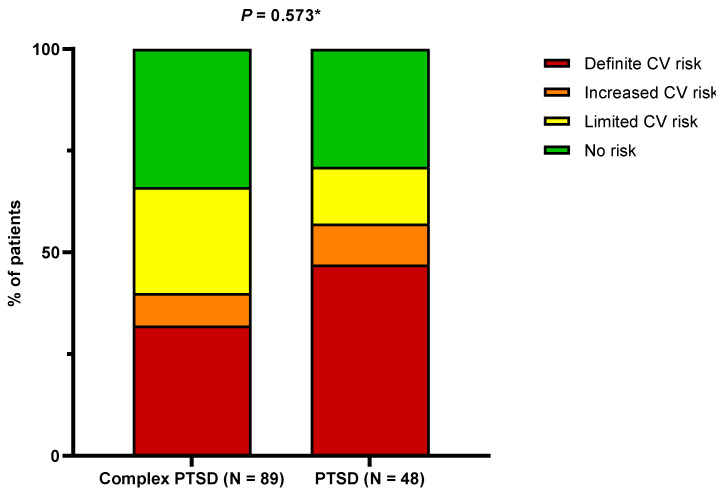
Differences in cardiovascular risk, as estimated by AGEs, with respect to the presence of the complex form of PTSD. Abbreviations: AGEs: advanced glycation end products; CV: cardiovascular; PTSD: post-traumatic stress disorder. * Chi-squared test.

Generally, a significant proportion of the studied population demonstrated “definite” CV risk (39.7%). No difference in CV risk, calculated based on AGEs, was found between patients with PTSD and complex PTSD (*p* = 0.573) (Figure 2). Furthermore, the tissue levels of AGEs did not differ with respect to the terciles of the total MDSS (2.4 (2.2–3.0) vs. 2.8 (2.4–3.5) vs. 2.4 (2.2–3.3), *p* = 0.407). Accordingly, no correlation was found between total MDSS and AGE levels (r = 0.070, *p* = 0.601).

### 3.3. Complex PTSD Has Profound Impact on Lifestyle

**Figure 3:**
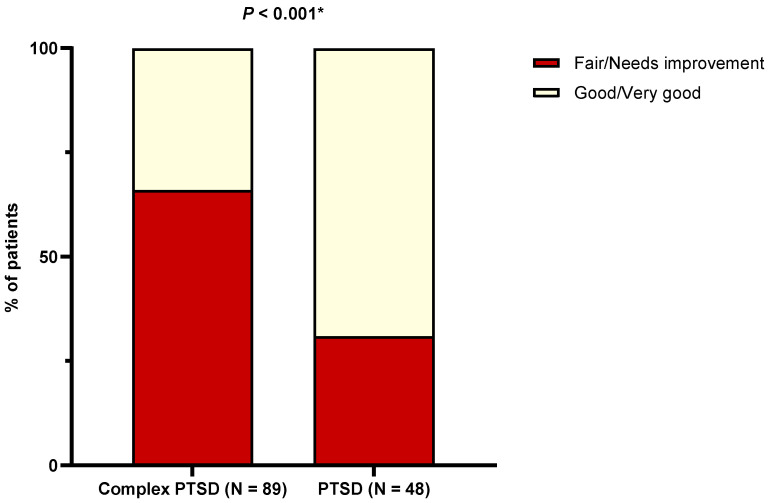
Comparisons in FLQ categories with respect to the presence of complex PTSD. Abbreviations: FLQ: Fantastic Lifestyle Questionnaire; PTSD: post-traumatic stress disorder. * Chi-squared test.

Patients with complex PTSD were shown to have significantly lower healthy lifestyle scores in comparison to PTSD counterparts (50.6 ± 9.7 vs. 59.6 ± 10.1, *p* < 0.001). Accordingly, patients with complex PTSD were far less likely to exhibit “good” or “very good” FLQ scores (Figure 3). Of note, no patients had an FLQ score that was considered “excellent” (≥ 85). Moreover, a weak positive correlation was found between total MDSS and the FLQ score (r = 0.183, *p* = 0.022). In fact, multivariate analysis showed that the two are associated independently of age, BMI, and complex PTSD status (β ± SE, 0.06 ± 0.02, *p* = 0.037).

### 3.4. Mediterranean Diet Adherence Does Not Appear to Affect Anthropometric Parameters or Glucose and Lipid Metabolism Indices

**Figure 4:**
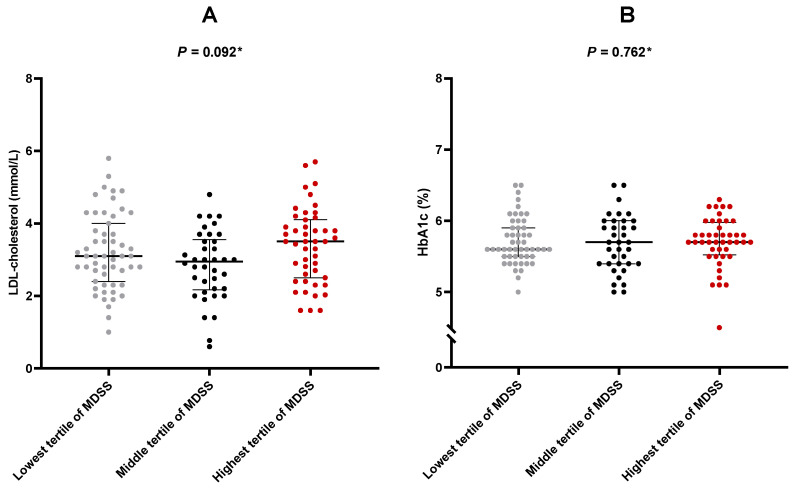
Comparison of LDL-cholesterol and HbA1c values with respect to MDSS terciles: (**A**) LDL-cholesterol; (**B**) HbA1c. Abbreviations: HbA1c: hemoglobin A1c; LDL: low-density lipoprotein; MDSS: Mediterranean Diet Serving Score. Dots represent individual values, large horizontal line represents median, whereas smaller lines represent 25th and 75th percentile, respectively. * The Kruskal–Wallis test.

No correlation was found between total MDSS and body fat percentage (r = −0.050, *p* = 0.571), visceral fat (r = −0.049, *p* = 0.572), muscle mass (r = 0.155, *p* = 0.333), or phase angle (r = −0.056, *p* = 0.525). Similarly, no difference was found in HbA1c or LDL-cholesterol between MDSS terciles (*p* = 0.728 and *p* = 0.092, respectively) (Figure 4).

### 3.5. The Presence of Complex PTSD Does Not Appear to Affect Physical Activity

**Figure 5:**
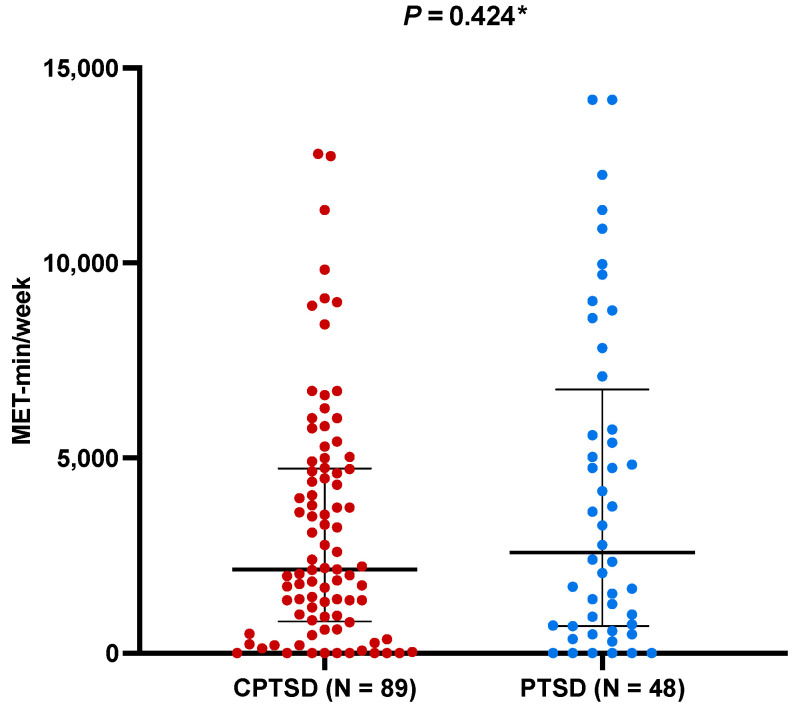
Comparison between physical activity depending on the presence of complex PTSD. Abbreviations: PTSD: post-traumatic stress disorder. Dots represent individual values, large horizontal line represents median, whereas smaller lines represent 25th and 75th percentile, respectively. * The Mann–Whitney test.

Finally, no difference was found in physical activity between patients with PTSD and patients with complex PTSD (2586 (702–6414) MET-min/week vs. 2148 (828–4724) MET-min/week, *p* = 0.424) (Figure 5). In addition, no correlation was found between physical activity and MDSS adherence (r = 0.340, *p* = 0.693) or FLQ score (r = 0.135, *p* = 0.117).

## 4. Discussion

To the best of our knowledge, this study constitutes the first report of a comprehensive lifestyle and CV risk comparison between patients with PTSD and complex PTSD. Specifically, conforming to the contemporary ICD-11 criteria, we demonstrated that war veterans with complex PTSD had lower healthy lifestyle levels and adherence to the Mediterranean diet. However, these alterations did not seem to translate to their cholesterol and HbA1c levels, physical activity, body composition or CV risk as assessed by AGEs.

That increased prevalence of an unhealthy lifestyle in patients with PTSD is even more pronounced in complex PTSD is an unsurprising result. Indeed, clusters of symptoms that burden PTSD patients, such as sleep disturbance, flashbacks, and anxiety, were previously shown to substantially reduce quality of life [21,22,23,24,25]. While a limited number of studies have formally examined the differences in quality of life depending on the presence of DSO symptoms, the available results consistently indicate a correlation between poor quality of life and complex PTSD [26,27,28]. Apart from the poor quality of life, which is inherently linked to unhealthy lifestyle practices, particularly concerning sleep hygiene and stress coping, the authors believe that many of these unhealthy habits originated during wartime, marked by a notable surge in the consumption of tobacco, alcohol, and illicit drugs.

The present study further substantiates the notion that MD adherence is exceedingly low in population, even in Mediterranean countries [12,29,30]. Furthermore, we demonstrated that MD adherence is even lower in patients with DSO symptoms. A bidirectional relationship seems to exist between MD and mental health. Specifically, due to the pronounced burden of stress, anxiety, and depression, patients with PTSD are more likely to neglect healthy dietary patterns and instead indulge in foods abundant in processed sugars. On the other hand, previous research has indicated that following the MD pattern may in fact alleviate PTSD symptoms, possibly by affecting the gut–brain axis [31]. Specifically, several species such as *Eubacterium eligens* and *Akkermansia muciniphila* and their functions were identified as “protective” since they were associated with fewer PTSD symptoms. The study also revealed a correlation between the quantity of PTSD symptoms and reduced adherence to MD, particularly concerning the inadequate consumption of plant-based foods, aligning with the findings of this investigation [31]. Indeed, in our analysis, the greatest difference between groups was observed in terms of adherence to vegetables, as less than 5% of complex PTSD patients were considered adherent. Although adhering to MD was reliably shown to contribute to weight and fat loss in previous research, we found no differences in indices of body composition, LDL-cholesterol or HbA1c between patients with PTSD and complex PTSD, as well as a lack of association between adherence to MD and these indices [32,33]. Furthermore, despite worse indices of healthy lifestyle and an increased mental health burden that theoretically should lead to reduced physical activity, patients with complex PTSD exhibited similar levels of physical activity as PTSD patients without DSO symptoms. Without reliable information concerning total daily caloric intake, and in light of similar physical activity, it is possible that differences in dietary pattern were not sufficient to translate to body weight and fat mass reduction in PTSD patients. Nonetheless, based on the available data, we can make an educated guess that we would spot differences in body weight, blood sugar and cholesterol levels during the follow-up of these patients. Our observation that both PTSD and complex PTSD groups are moderately active according to IPAQ criteria contrasts with other studies that have documented lower levels of physical activity among individuals with PTSD [34,35]. This discrepancy could be attributed to several factors unique to our study cohort, including demographic characteristics, recruitment methods, or regional differences in lifestyle and healthcare access. Importantly, as regular physical activity is known to confer numerous health benefits, including improved cardiovascular health, better metabolic control, and enhanced mental well-being, the moderate activity levels observed in our study participants could serve as a protective factor. This may help to explain the lack of significant differences in cholesterol levels, HbA1c levels, body composition, and cardiovascular risk as measured by AGEs, despite the complex PTSD group having poorer adherence to the Mediterranean diet and a generally less healthy lifestyle. Thus, this finding underscores the importance of considering physical activity to be a variable that can influence health-related outcomes in PTSD research. It suggests that promoting physical activity among individuals with PTSD could be a valuable intervention strategy to improve their overall health [36]. Future studies should explore the mechanisms through which physical activity impacts health outcomes in PTSD populations and investigate whether specific types of physical activity are more beneficial. Regarding the positive and independent association observed between adherence to the MD and FLQ, we contend that this information further validates the notion that FLQ is indeed a valuable proxy for multiple aspects of a healthy lifestyle, encompassing dietary habits. 

Numerous population-based studies have consistently demonstrated that PTSD patients are burdened by increased CVD risk [37]. Except for indirect effects of lifestyle choices, multiple authors discussed the direct relationship between heightened CV risk and psychological stress in the PTSD population [38,39,40]. A commonly reported denominator between psychological stress and CV disease is disturbance in pro-oxidant/antioxidant balance, although the causal relationship between the two has not been fully elucidated [41]. Some authors even indicated that increasing the intensity and/or chronicity of experienced trauma underlies more prominent mitochondrial dysfunction with consequent cell damage, resulting in accelerated immunosenescence, neuroprogression, and neurodegeneration [42]. In the setting of PTSD, Borovac et al. reported that in patients exposed to war-related trauma and/or PTSD diagnosis, the activity of antioxidants is reduced, whereas the production of reactive nitrogen species is increased [43]. Accordingly, patients with PTSD exhibit dysfunctional baroreceptor reflex, reduced heart rate variability, and increased QT variability, all of which have been linked to increased CVD risk [44,45,46]. The observed absence of disparity in estimated CV risk between PTSD and complex PTSD is challenging to interpret. Since physical inactivity, poor dietary preferences, and less healthy lifestyle are robust indicators of heightened CV risk, the observation that AGE-based CV risk did not vary between PTSD and complex PTSD does not definitively suggest a lack of difference in CV risk between these patients. Rather, it may raise questions about the sensitivity of AGEs as a method for estimating CV risk in this population. Indeed, if heightened oxidative stress indeed associates PTSD with poor CV outcomes, AGEs should theoretically constitute a representative tool to explore it, given that AGEs increasingly accumulate in states of pro-oxidant/antioxidant imbalance [47,48]. In summary, as we only cross-sectionally examined AGEs between patients with PTSD and complex PTSD, it remains elusive if these subgroups indeed have comparable CV risks (elevated but to a similar extent) or AGE tissue levels are not sensitive enough to demonstrate the difference.

This study has several limitations worth noting. Firstly, its cross-sectional design prohibits us from achieving the establishment of a causal relationship. The fact that only treated male war veterans with PTSD were included limits the generalization of our results to other populations (female, PTSD unrelated to war, untreated PTSD population). Accordingly, the inclusion of healthy matched controls without PTSD would provide valuable insight. Although tissue AGEs levels are a well-validated CV risk assessment tool, they represent only one aspect of CV risk, and it is, therefore, possible that some patients were not adequately stratified. Finally, the self-administered format of the questionnaires used in this study might introduce bias and unreliable responses.

## 5. Conclusions

Altogether, the present research compares the difference in multiple aspects of lifestyle and CV risk between PTSD and complex PTSD patients. In concordance with the data from healthy population, overall MD adherence was very low in PTSD patients, reflecting the shift towards a Westernized diet even in Mediterranean basin countries. Despite the fact that lower quality of life and poor dietary choices were more prevalent in complex PTSD patients, no difference was found in physical activity, body composition, or estimated CV risk. Furthermore, a positive association was noted between a healthy lifestyle and Mediterranean diet adherence, indicating that patients with healthier lifestyles are more likely to make appropriate dietary choices. As dietary choices and lifestyle are well established CV risks, it remains to be determined in future research if our results in terms of CV risk reflect the real state of the PTSD population or lack the power to demonstrate difference.

## Figures and Tables

**Table 1:** The baseline characteristics of the studied population.

Parameter	Total Population (*n* = 137)	PTSD Group (*n* = 48)	Complex PTSD Group (*n* = 89)	*p* *
Age, years	57.1 ± 5.6	57.5 ± 6.2	56.9 ± 5.4	0.559
Body mass index, kg/m^2^	28.4 ± 4.0	28.3 ± 4.3	28.5 ± 3.8	0.862
Waist-to-hip ratio	0.97 ± 0.06	0.95 ± 0.06	0.98 ± 0.06	0.095
Body fat, %	26.1 ± 5.2	26.4 ± 5.4	26.0 ± 5.1	0.692
Visceral fat, %	13.4 ± 3.4	13.7 ± 3.9	13.2 ± 3.2	0.478
Phase angle, °	5.5 ± 1.4	5.5 ± 1.2	5.5 ± 1.5	0.938
Hemoglobin A1c, %	5.8 ± 0.4	5.7 ± 0.4	5.8 ± 0.4	0.309
Total cholesterol, mmol/L	5.4 ± 1.2	5.6 ± 1.3	5.3 ± 1.1	0.100
LDL-C, mmol/L	3.2 ± 1.0	3.4 ± 1.1	3.1 ± 1.0	0.184
HDL-C, mmol/L	1.2 (1.0–1.5)	1.3 (1.1–1.6)	1.2 (1.0–1.4)	0.266
Triglycerides, mmol/L	1.5 (1.1–2.2)	1.4 (1.0–1.8)	1.7 (1.1–2.3)	0.284
Smoking, *n* (%)	61 (44.5)	18 (37.5)	43 (48.3)	0.226
Alcohol **, *n* (%)	76 (55.5)	30 (62.5)	46 (51.7)	0.226
Hypertension, *n* (%)	88 (64.2)	30 (62.5)	58 (65.2)	0.757
Socioeconomic status, *n* (%)				
Low	51 (37.2)	13 (27.1)	38 (42.7)	0.174
Average	6 (4.4)	32 (66.7)	3 (3.4)	
Above average	80 (58.4)	3 (6.2)	48 (53.9)	
Work status, *n* (%)				
Employed	45 (32.8)	11 (22.9)	31 (34.8)	**0.007**
Unemployed	42 (30.7)	11 (22.9)	34 (38.2)	
Retired	50 (36.5)	26 (54.2)	24 (27.0)	
Education, *n* (%)				
Elementary school	23 (16.8)	10 (20.8)	13 (14.6)	
High school	98 (71.5)	32 (66.7)	66 (74.2)	0.604
Higher education	16 (11.7)	6 (12.5)	10 (11.2)	
Treatment duration, years	12.8 ± 9.7	12.3 ± 9.3	13.0 ± 9.9	0.682
Time spent in war, years	2.4 (1.0–3.5)	2.6 (1.4–4.5)	1.9 (1.0–1.3)	**0.034**

Data presented as mean ± SD, median (IQR), or *n* (%). * Welch’s *t*-test, the Mann–Whitney U test or the chi-squared test, as appropriate. ** Regular alcohol consumption. Bolded values indicate significant difference between the groups. Abbreviations: HDL-C: high-density lipoprotein cholesterol; LDL-C: low-density lipoprotein cholesterol; PTSD: post-traumatic stress disorder.

**Table 2:** Adherence to individual food groups of the Mediterranean diet according to the presence of the complex form of PTSD.

Parameter	Total Population (*n* = 137)	PTSD Group (*n* = 48)	CPTSD Group (*n* = 89)	*p* *
Cereals, *n* (%)	66 (48.2)	26 (54.2)	40 (45.0)	0.304
Potato, *n* (%)	108 (78.8)	38 (79.2)	70 (78.7)	0.944
Olive oil, *n* (%)	22 (16.1)	8 (16.7)	14 (15.7)	0.887
Nuts, *n* (%)	24 (17.5)	9 (18.8)	15 (16.9)	0.781
Fresh fruit, *n* (%)	16 (11.7)	5 (10.4)	11 (12.4)	0.736
Vegetables, *n* (%)	16 (11.7)	12 (25.0)	4 (4.5)	**<0.001**
Milk and dairy products, *n* (%)	24 (17.5)	7 (14.6)	17 (19.1)	0.509
Legumes, *n* (%)	13 (9.5)	4 (8.3)	9 (10.1)	0.736
Eggs, *n* (%)	70 (51.1)	23 (47.9)	47 (52.8)	0.586
Fish, *n* (%)	97 (70.8)	35 (72.9)	62 (69.7)	0.691
White meat, *n* (%)	49 (35.8)	12 (25.0)	37 (41.6)	0.054
Red meat, *n* (%)	34 (24.8)	12 (25.0)	22 (24.7)	0.971
Sweets, *n* (%)	65 (47.4)	21 (43.8)	44 (49.4)	0.526
Wine, *n* (%)	44 (32.1)	19 (39.6)	25 (28.1)	0.171

Data presented as *n* (%). * Chi-squared test or Fisher’s exact test. Bolded values indicate significant difference between the groups. Abbreviations: PTSD: post-traumatic stress disorder.

## Data Availability

The data that were presented in this study are available on request from the corresponding author. The data are not accessible to the public due to ethical constraints.
